# Could unpublishing negative results be harmful to the general public?

**DOI:** 10.2478/jtim-2023-0120

**Published:** 2023-12-20

**Authors:** Katrin Sak

**Affiliations:** NGO Praeventio, Tartu 50407, Estonia

In biomedicine, the question of publishing negative results showing no remarkable effects on the targets of interest (*i. e*., no evidence of an effect) has always been a scientific dilemma to both scientists as well as publishers, due to a widespread view that readers generally wait for positive results that seemingly make the things better.^[1]^ In this short commentary article, an important aspect in favor of publishing also non-significant findings is presented, highlighting the necessity to share negative scientific information as well.

The paucity of reports of investigations with negative or non-significant results in medical journals was noticed already in 1997, bringing forth the editors` preference to publish predominantly articles with significant, positive results.^[2]^ The price of such a choice is a high probability that the same experiments will shortly be repeated by other researchers, a process that is related to the waste of time, money and needless effort. This is especially disturbing in the case of clinical trials involving patients who will undergo an intervention which effect is previously found to be non-significant, but being unpublished, not shared with the overall scientific community.^[2]^ Other convincing arguments in favor of publishing negative results include the ethical commitment to study participants (both humans and animals) to make the results publicly available, besides giving valuable input to systematic reviews for presenting a complete picture of the subject.^[3]^ In this way, there might be an abundance of negative results with positive messages and learning experiences that could give an important impulse for the scientific progression by raising and testing new attractive hypotheses.^[4]^ In short, to know what works, we should also understand what (and why) does not work.^[1]^

In addition to these previously presented arguments, publishing negative results might also decrease the production of false-positive conclusions from meta-analyses. In fact, considering epidemiological prospective cohort studies, the finding of no significant association between a factor under investigation (for example, the dietary intake of specific compounds or food products rich in these compounds) and relevant clinical endpoints (for example, morbidity or mortality rate of certain diseases) may indicate the true lack of relationship in a particular study population or non-adjustment for some important confounding parameters. Being an apparent disappointment, leaving such results unpublished can still lead to more severe consequences. Indeed, the implementation of large-scale meta-analyses which make thorough conclusions about the strength of correlations is usually based on the published results of observational investigations. It is not difficult to understand that publishing only positive outcomes with statistically significant effects enhances the bias of meta-analyses, possibly leading to inadequate conclusions and giving unrealistic information to the general public, for example concerning the health benefits of specific dietary compounds/food products towards the risk of serious diseases. Besides a possible false-positivity, sharing such a knowledge among general population may involve a wider and useless consumption of an ineffective product with largely unknown safety profile and potentially harmful side effects. Therefore, it is important to encourage publishing also those results that reveal no or statistically non-significant associations between parameters under consideration, along with a detailed description of study populations and adjusted confounding factors. In this context, the large number of recruited subjects (and a considerable number of cases) cannot necessarily ensure clinical relevance of meta-analyses, even when presented with a sufficient statistical power, if the involved data are initially biased and the unpublished negative results are unknowingly excluded. This argument speaks strongly for publishing also the reports of investigations with non-significant, negative results. To give more support to this process, it is needed to change the negative psychological view on non-significant scientific findings, as negative results are not yet the feature of a bad science.

Comprehending the huge amount of data with non-significant findings produced every day in experimental settings around the world, the development of some general criteria for evaluating the relevance of such results to the scientific and medical progression seems to be justified. This involves first the actual definition of “negative results”, as reproducible findings of a well-planned experiment should probably not be considered truly negative, even when showing non-significant associations between the study parameters. A fundamental factor in appreciating any experimental results is their high reproducibility in different biological systems, whereas some non-significant findings proven in several *in vitro* and *in vivo* models may be more informative than positive results observed only in a single cell line. Secondly, an overall approach to non-significant findings could primarily proceed from the Latin phrase *primum non nocere* (first, do not harm), implying that all the findings revealing any potential harm should be made public and share with the general community. This concerns especially the results from large-scale epidemiological studies and clinical trials, where unpublishing non-significant associations may lead to the false-positive conclusions from meta-analyses, a possibility described more closely in this commentary article.
